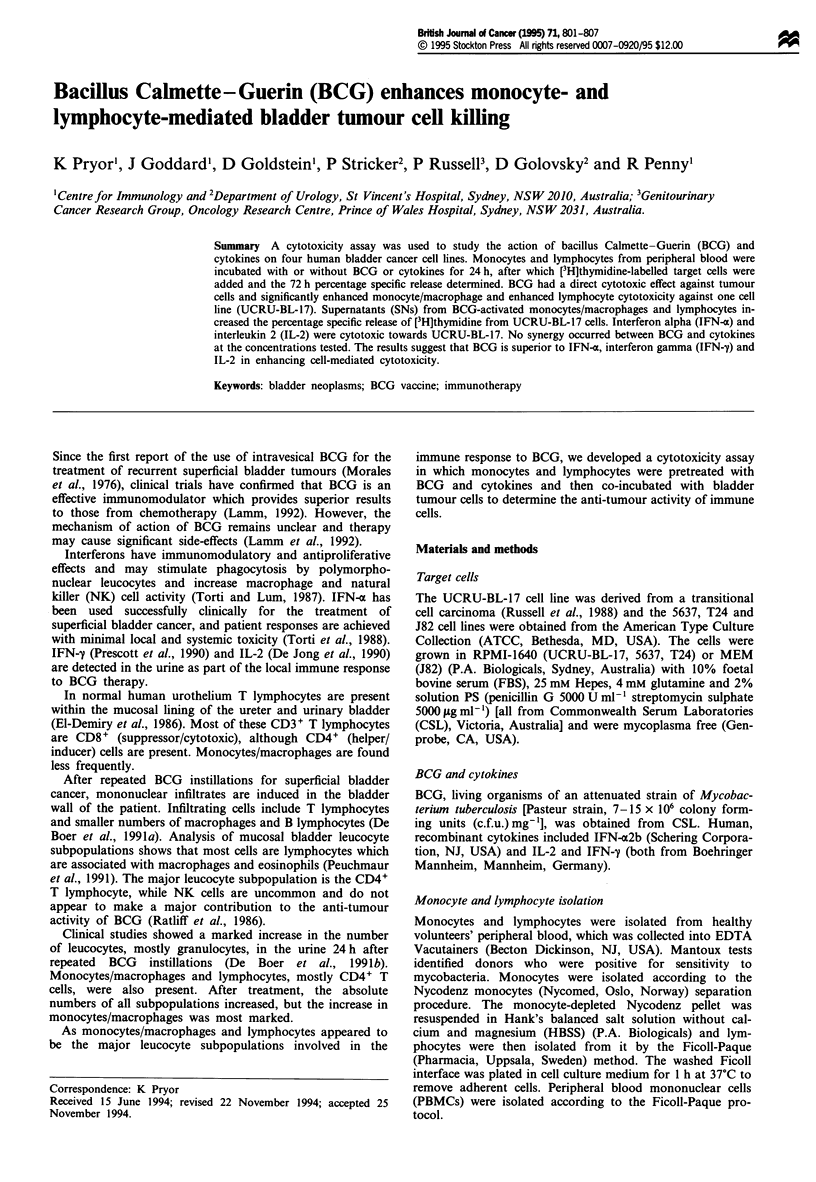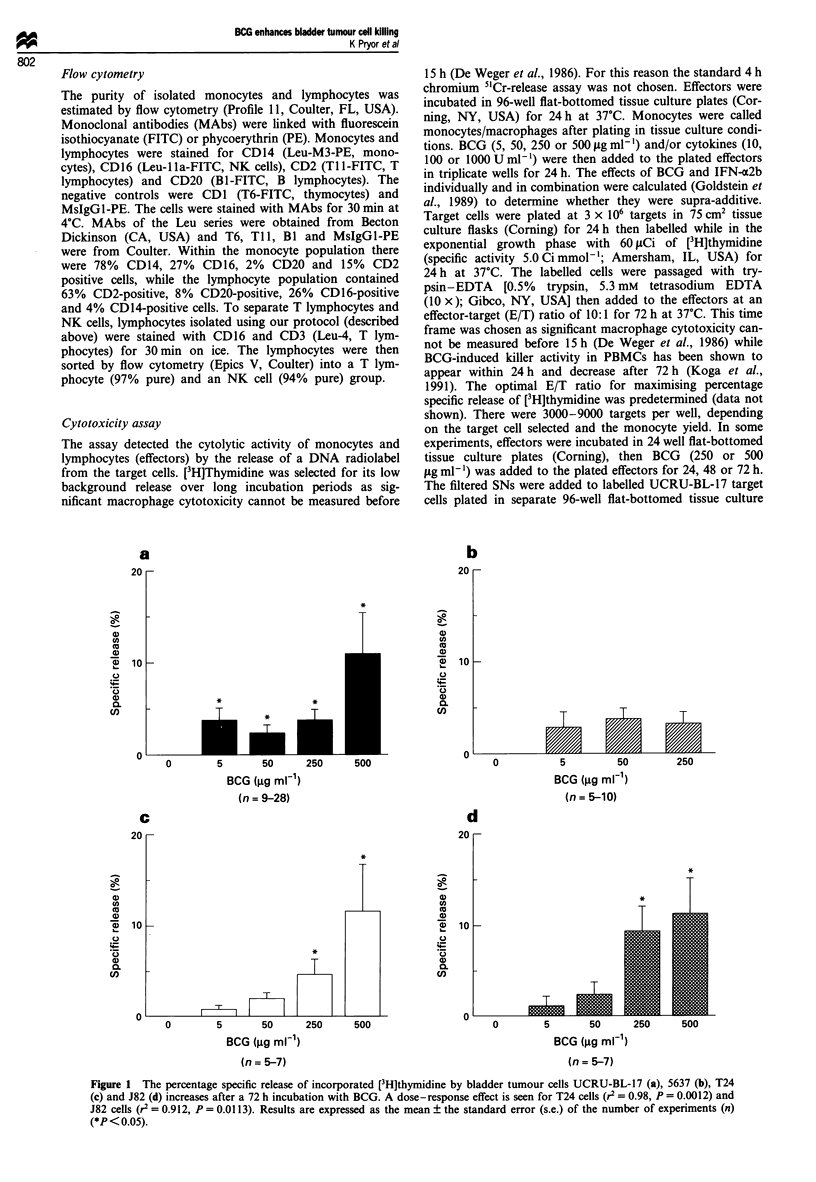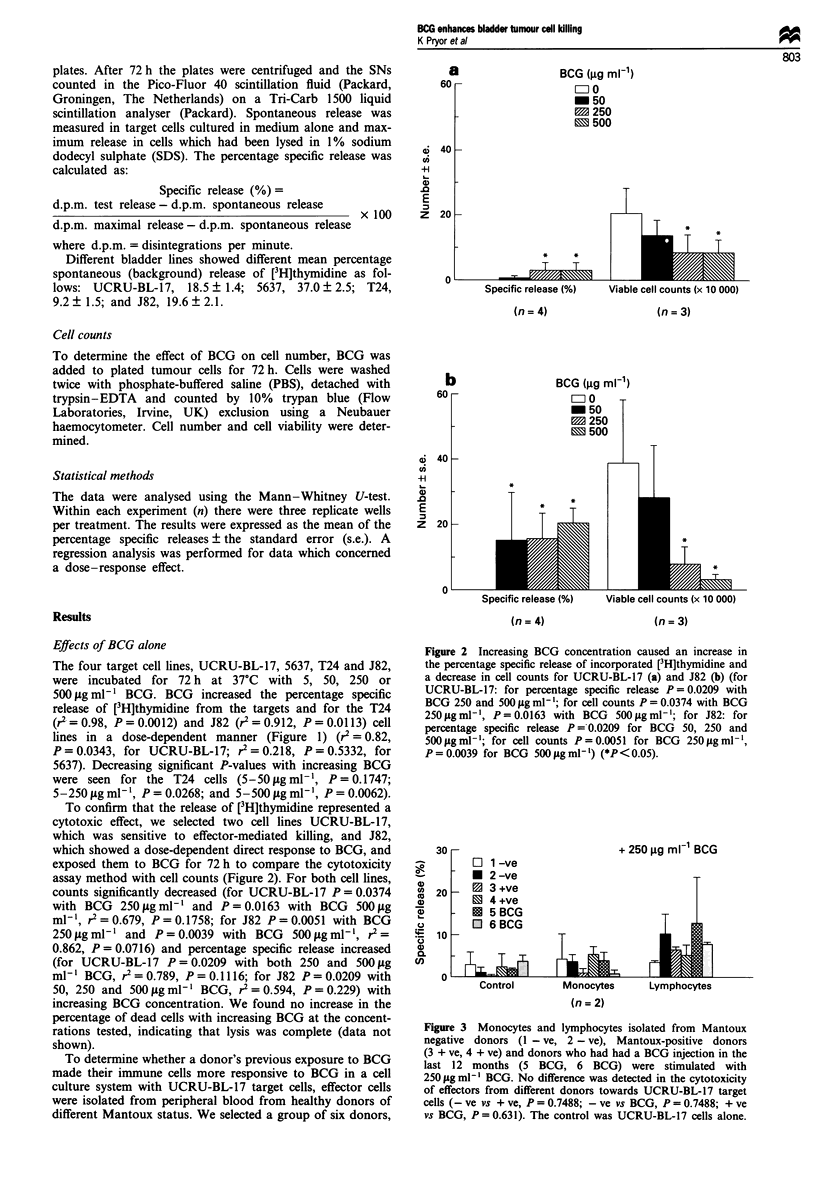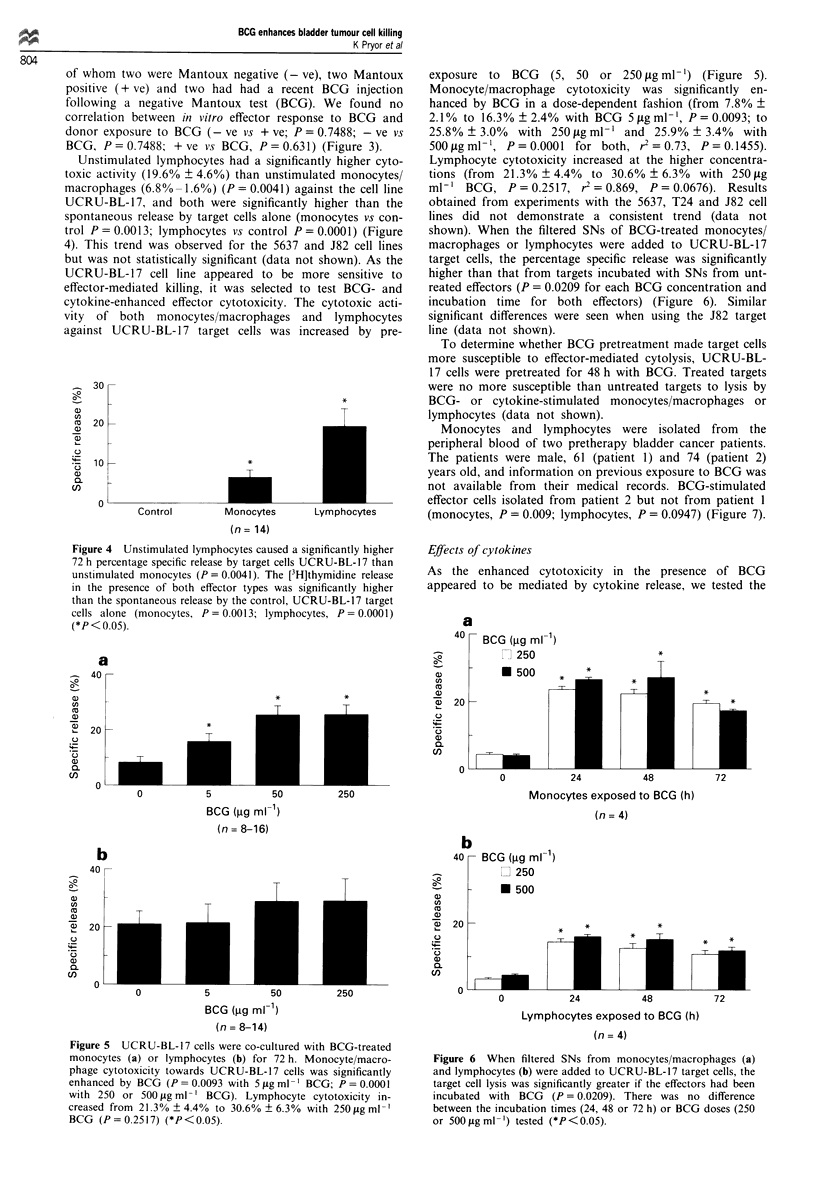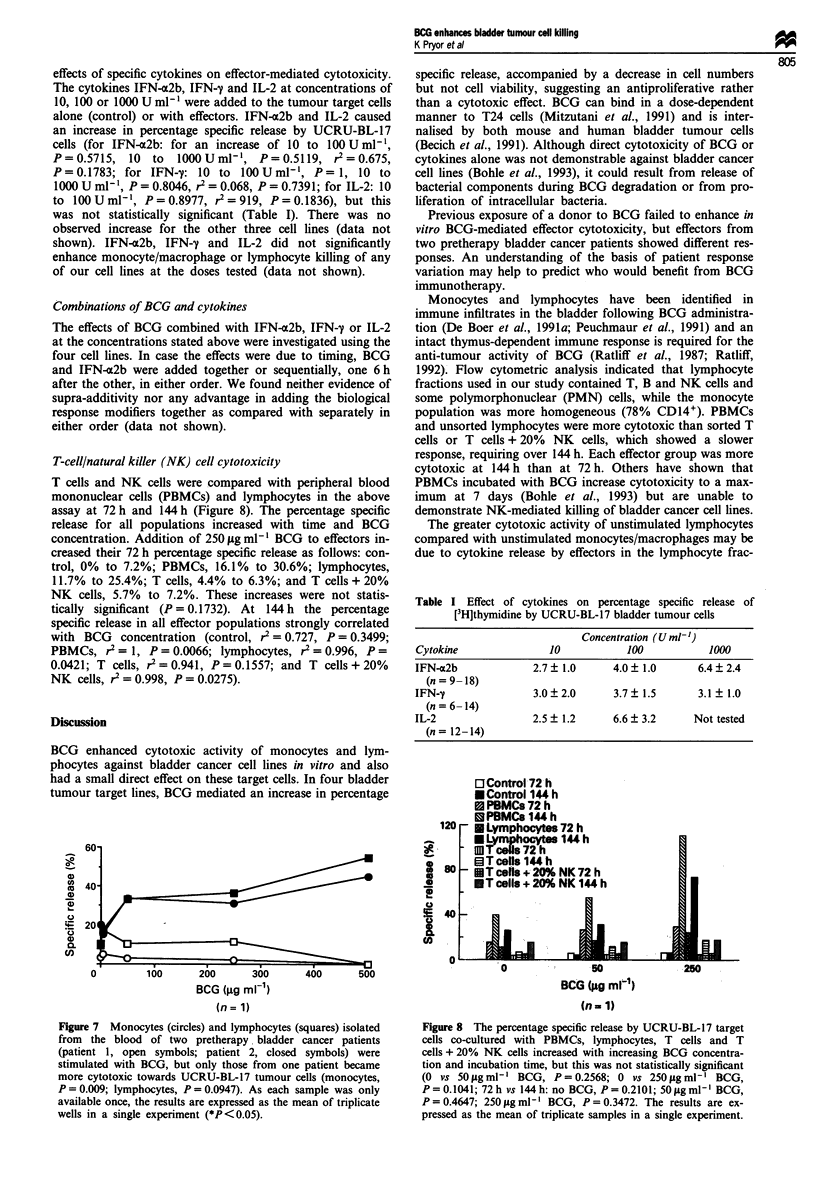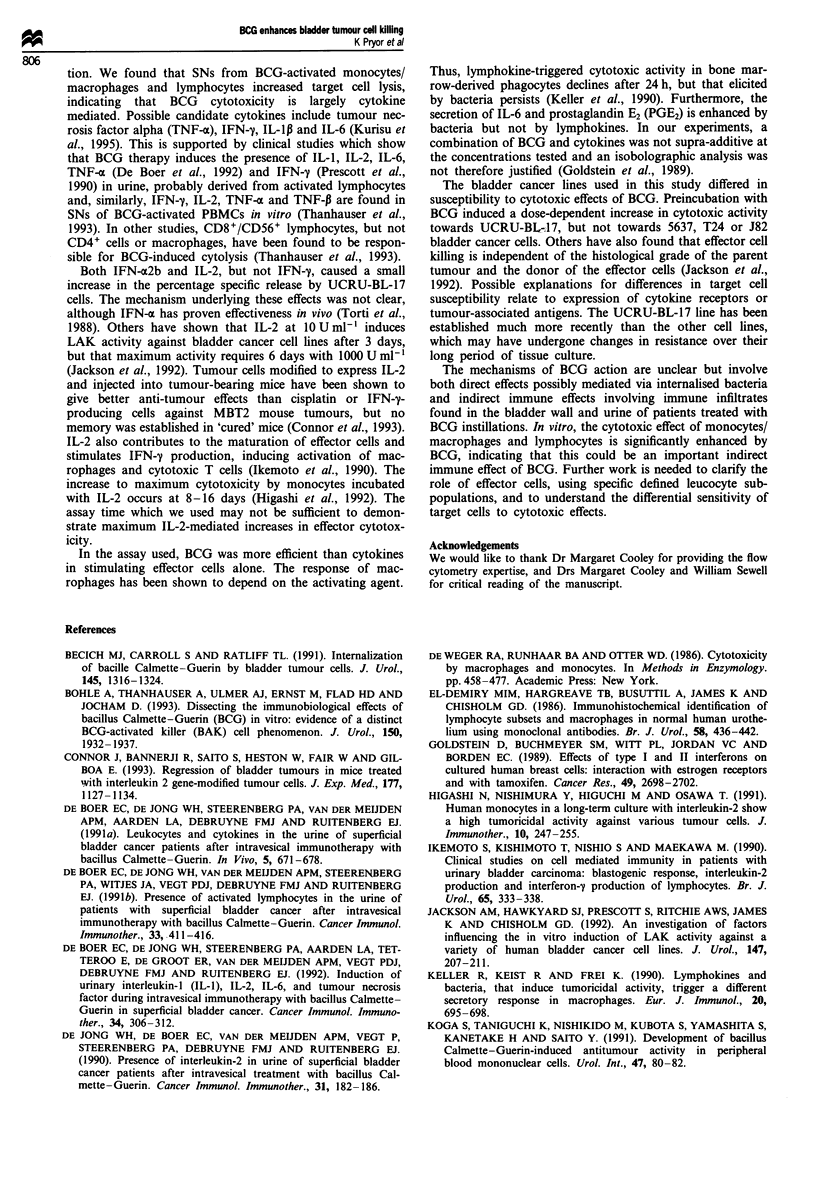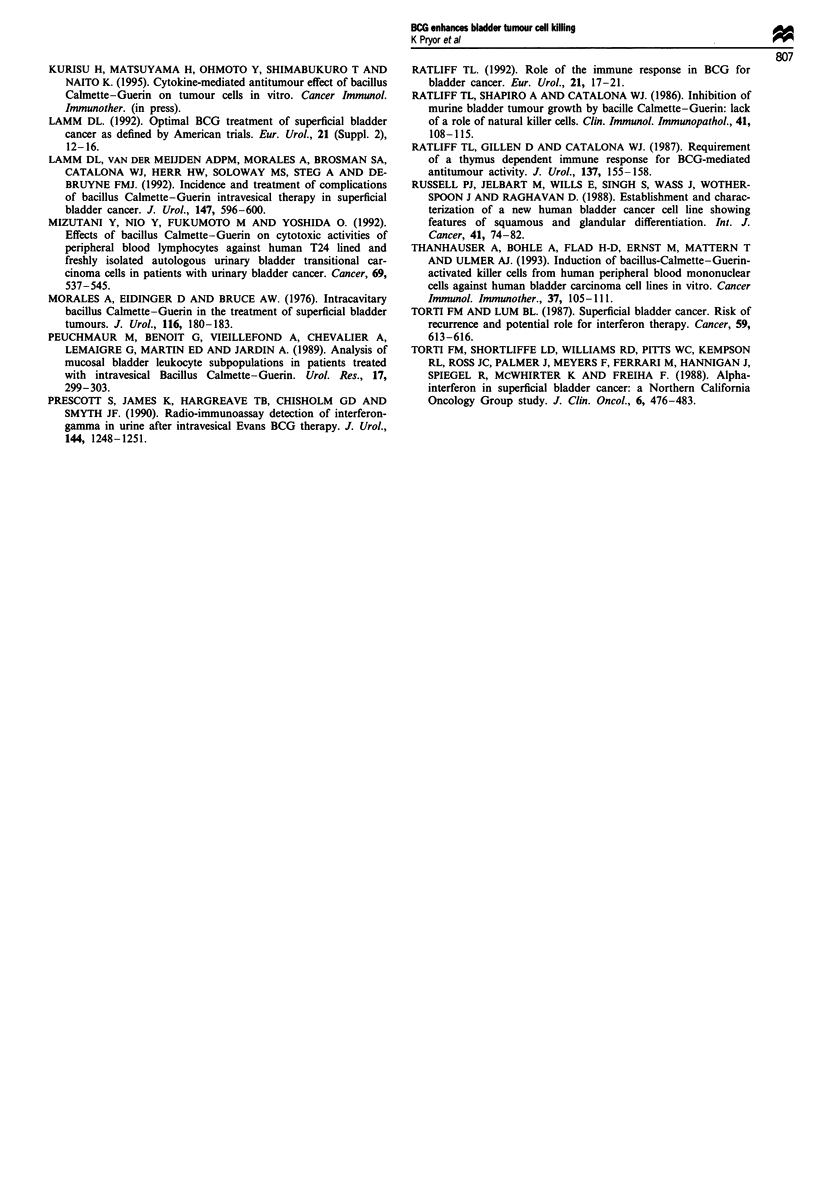# Bacillus Calmette-Guerin (BCG) enhances monocyte- and lymphocyte-mediated bladder tumour cell killing.

**DOI:** 10.1038/bjc.1995.155

**Published:** 1995-04

**Authors:** K. Pryor, J. Goddard, D. Goldstein, P. Stricker, P. Russell, D. Golovsky, R. Penny

**Affiliations:** Centre for Immunology, St Vincent's Hospital, Sydney, NSW, Australia.

## Abstract

A cytotoxicity assay was used to study the action of bacillus Calmette-Guerin (BCG) and cytokines on four human bladder cancer cell lines. Monocytes and lymphocytes from peripheral blood were incubated with or without BCG or cytokines for 24 h, after which [3H]thymidine-labelled target cells were added and the 72 h percentage specific release determined. BCG had a direct cytotoxic effect against tumour cells and significantly enhanced monocyte/macrophage and enhanced lymphocyte cytotoxicity against one cell line (UCRU-BL-17). Supernatants (SNs) from BCG-activated monocytes/macrophages and lymphocytes increased the percentage specific release of [3H]thymidine from UCRU-BL-17 cells. Interferon alpha (IFN-alpha) and interleukin 2 (IL-2) were cytotoxic towards UCRU-BL-17. No synergy occurred between BCG and cytokines at the concentrations tested. The results suggest that BCG is superior to IFN-alpha, interferon gamma (IFN-gamma) and IL-2 in enhancing cell-mediated cytotoxicity.


British Journal of Cancer (1995) 71, 801-807
© 1995 Stockton Press All rights reserved 0007-0920/95 $12.00

### Bacillus Calmette–Guerin (BCG) enhances monocyte- and lymphocyte-mediated bladder tumour cell killing

K Pryor[1], J Goddard[1], D Goldstein[1], P Stricker[2], P Russell[3], D Golovsky[2] and R Penny[1]

[1]*Centre for Immunology and* [2]*Department of Urology, St Vincent's Hospital, Sydney, NSW 2010, Australia;* [3]*Genitourinary Cancer Research Group, Oncology Research Centre, Prince of Wales Hospital, Sydney, NSW 2031, Australia.*

**Summary** A cytotoxicity assay was used to study the action of bacillus Calmette–Guerin (BCG) and cytokines on four human bladder cancer cell lines. Monocytes and lymphocytes from peripheral blood were incubated with or without BCG or cytokines for 24 h, after which [$^3$H]thymidine-labelled target cells were added and the 72 h percentage specific release determined. BCG had a direct cytotoxic effect against tumour cells and significantly enhanced monocyte/macrophage and enhanced lymphocyte cytotoxicity against one cell line (UCRU-BL-17). Supernatants (SNs) from BCG-activated monocytes/macrophages and lymphocytes increased the percentage specific release of [$^3$H]thymidine from UCRU-BL-17 cells. Interferon alpha (IFN-$\alpha$) and interleukin 2 (IL-2) were cytotoxic towards UCRU-BL-17. No synergy occurred between BCG and cytokines at the concentrations tested. The results suggest that BCG is superior to IFN-$\alpha$, interferon gamma (IFN-$\gamma$) and IL-2 in enhancing cell-mediated cytotoxicity.

**Keywords**: bladder neoplasms; BCG vaccine; immunotherapy

Since the first report of the use of intravesical BCG for the treatment of recurrent superficial bladder tumours (Morales *et al.*, 1976), clinical trials have confirmed that BCG is an effective immunomodulator which provides superior results to those from chemotherapy (Lamm, 1992). However, the mechanism of action of BCG remains unclear and therapy may cause significant side-effects (Lamm *et al.*, 1992).

Interferons have immunomodulatory and antiproliferative effects and may stimulate phagocytosis by polymorphonuclear leucocytes and increase macrophage and natural killer (NK) cell activity (Torti and Lum, 1987). IFN-$\alpha$ has been used successfully clinically for the treatment of superficial bladder cancer, and patient responses are achieved with minimal local and systemic toxicity (Torti *et al.*, 1988). IFN-$\gamma$ (Prescott *et al.*, 1990) and IL-2 (De Jong *et al.*, 1990) are detected in the urine as part of the local immune response to BCG therapy.

In normal human urothelium T lymphocytes are present within the mucosal lining of the ureter and urinary bladder (El-Demiry *et al.*, 1986). Most of these CD3$^+$ T lymphocytes are CD8$^+$ (suppressor/cytotoxic), although CD4$^+$ (helper/inducer) cells are present. Monocytes/macrophages are found less frequently.

After repeated BCG instillations for superficial bladder cancer, mononuclear infiltrates are induced in the bladder wall of the patient. Infiltrating cells include T lymphocytes and smaller numbers of macrophages and B lymphocytes (De Boer *et al.*, 1991*a*). Analysis of mucosal bladder leucocyte subpopulations shows that most cells are lymphocytes which are associated with macrophages and eosinophils (Peuchmaur *et al.*, 1991). The major leucocyte subpopulation is the CD4$^+$ T lymphocyte, while NK cells are uncommon and do not appear to make a major contribution to the anti-tumour activity of BCG (Ratliff *et al.*, 1986).

Clinical studies showed a marked increase in the number of leucocytes, mostly granulocytes, in the urine 24 h after repeated BCG instillations (De Boer *et al.*, 1991*b*). Monocytes/macrophages and lymphocytes, mostly CD4$^+$ T cells, were also present. After treatment, the absolute numbers of all subpopulations increased, but the increase in monocytes/macrophages was most marked.

As monocytes/macrophages and lymphocytes appeared to be the major leucocyte subpopulations involved in the immune response to BCG, we developed a cytotoxicity assay in which monocytes and lymphocytes were pretreated with BCG and cytokines and then co-incubated with bladder tumour cells to determine the anti-tumour activity of immune cells.

Correspondence: K Pryor
Received 15 June 1994; revised 22 November 1994; accepted 25 November 1994.

### Materials and methods

### *Target cells*

The UCRU-BL-17 cell line was derived from a transitional cell carcinoma (Russell *et al.*, 1988) and the 5637, T24 and J82 cell lines were obtained from the American Type Culture Collection (ATCC, Bethesda, MD, USA). The cells were grown in RPMI-1640 (UCRU-BL-17, 5637, T24) or MEM (J82) (P.A. Biologicals, Sydney, Australia) with 10% foetal bovine serum (FBS), 25 mM Hepes, 4 mM glutamine and 2% solution PS (penicillin G 5000 U ml$^{-1}$ streptomycin sulphate 5000 $\mu$g ml$^{-1}$) [all from Commonwealth Serum Laboratories (CSL), Victoria, Australia] and were mycoplasma free (Genprobe, CA, USA).

### *BCG and cytokines*

BCG, living organisms of an attenuated strain of *Mycobacterium tuberculosis* [Pasteur strain, 7–15 × 10$^6$ colony forming units (c.f.u.) mg$^{-1}$], was obtained from CSL. Human, recombinant cytokines included IFN-$\alpha$2b (Schering Corporation, NJ, USA) and IL-2 and IFN-$\gamma$ (both from Boehringer Mannheim, Mannheim, Germany).

### *Monocyte and lymphocyte isolation*

Monocytes and lymphocytes were isolated from healthy volunteers' peripheral blood, which was collected into EDTA Vacutainers (Becton Dickinson, NJ, USA). Mantoux tests identified donors who were positive for sensitivity to mycobacteria. Monocytes were isolated according to the Nycodenz monocytes (Nycomed, Oslo, Norway) separation procedure. The monocyte-depleted Nycodenz pellet was resuspended in Hank's balanced salt solution without calcium and magnesium (HBSS) (P.A. Biologicals) and lymphocytes were then isolated from it by the Ficoll-Paque (Pharmacia, Uppsala, Sweden) method. The washed Ficoll interface was plated in cell culture medium for 1 h at 37°C to remove adherent cells. Peripheral blood mononuclear cells (PBMCs) were isolated according to the Ficoll-Paque protocol.

*Flow cytometry*

The purity of isolated monocytes and lymphocytes was estimated by flow cytometry (Profile 11, Coulter, FL, USA). Monoclonal antibodies (MAbs) were linked with fluorescein isothiocyanate (FITC) or phycoerythrin (PE). Monocytes and lymphocytes were stained for CD14 (Leu-M3-PE, monocytes), CD16 (Leu-11a-FITC, NK cells), CD2 (T11-FITC, T lymphocytes) and CD20 (B1-FITC, B lymphocytes). The negative controls were CD1 (T6-FITC, thymocytes) and MsIgG1-PE. The cells were stained with MAbs for 30 min at 4°C. MAbs of the Leu series were obtained from Becton Dickinson (CA, USA) and T6, T11, B1 and MsIgG1-PE were from Coulter. Within the monocyte population there were 78% CD14, 27% CD16, 2% CD20 and 15% CD2 positive cells, while the lymphocyte population contained 63% CD2-positive, 8% CD20-positive, 26% CD16-positive and 4% CD14-positive cells. To separate T lymphocytes and NK cells, lymphocytes isolated using our protocol (described above) were stained with CD16 and CD3 (Leu-4, T lymphocytes) for 30 min on ice. The lymphocytes were then sorted by flow cytometry (Epics V, Coulter) into a T lymphocyte (97% pure) and an NK cell (94% pure) group.

*Cytotoxicity assay*

The assay detected the cytolytic activity of monocytes and lymphocytes (effectors) by the release of a DNA radiolabel from the target cells. [$^3$H]Thymidine was selected for its low background release over long incubation periods as significant macrophage cytotoxicity cannot be measured before 15 h (De Weger *et al.*, 1986). For this reason the standard 4 h chromium $^{51}$Cr-release assay was not chosen. Effectors were incubated in 96-well flat-bottomed tissue culture plates (Corning, NY, USA) for 24 h at 37°C. Monocytes were called monocytes/macrophages after plating in tissue culture conditions. BCG (5, 50, 250 or 500 μg ml$^{-1}$) and/or cytokines (10, 100 or 1000 U ml$^{-1}$) were then added to the plated effectors in triplicate wells for 24 h. The effects of BCG and IFN-α2b individually and in combination were calculated (Goldstein *et al.*, 1989) to determine whether they were supra-additive. Target cells were plated at $3 \times 10^6$ targets in 75 cm$^2$ tissue culture flasks (Corning) for 24 h then labelled while in the exponential growth phase with 60 μCi of [$^3$H]thymidine (specific activity 5.0 Ci mmol$^{-1}$; Amersham, IL, USA) for 24 h at 37°C. The labelled cells were passaged with trypsin-EDTA [0.5% trypsin, 5.3 mM tetrasodium EDTA (10 ×); Gibco, NY, USA] then added to the effectors at an effector-target (E/T) ratio of 10:1 for 72 h at 37°C. This time frame was chosen as significant macrophage cytotoxicity cannot be measured before 15 h (De Weger *et al.*, 1986) while BCG-induced killer activity in PBMCs has been shown to appear within 24 h and decrease after 72 h (Koga *et al.*, 1991). The optimal E/T ratio for maximising percentage specific release of [$^3$H]thymidine was predetermined (data not shown). There were 3000–9000 targets per well, depending on the target cell selected and the monocyte yield. In some experiments, effectors were incubated in 24 well flat-bottomed tissue culture plates (Corning), then BCG (250 or 500 μg ml$^{-1}$) was added to the plated effectors for 24, 48 or 72 h. The filtered SNs were added to labelled UCRU-BL-17 target cells plated in separate 96-well flat-bottomed tissue culture

**Figure 1** The percentage specific release of incorporated [$^3$H]thymidine by bladder tumour cells UCRU-BL-17 (**a**), 5637 (**b**), T24 (**c**) and J82 (**d**) increases after a 72 h incubation with BCG. A dose-response effect is seen for T24 cells ($r^2 = 0.98$, $P = 0.0012$) and J82 cells ($r^2 = 0.912$, $P = 0.0113$). Results are expressed as the mean ± the standard error (s.e.) of the number of experiments (*n*) ($^*P < 0.05$).

plates. After 72 h the plates were centrifuged and the SNs counted in the Pico-Fluor 40 scintillation fluid (Packard, Groningen, The Netherlands) on a Tri-Carb 1500 liquid scintillation analyser (Packard). Spontaneous release was measured in target cells cultured in medium alone and maximum release in cells which had been lysed in 1% sodium dodecyl sulphate (SDS). The percentage specific release was calculated as:

$$\text{Specific release (\%)} = \frac{\text{d.p.m. test release} - \text{d.p.m. spontaneous release}}{\text{d.p.m. maximal release} - \text{d.p.m. spontaneous release}} \times 100$$

where d.p.m. = disintegrations per minute.

Different bladder lines showed different mean percentage spontaneous (background) release of [$^3$H]thymidine as follows: UCRU-BL-17, 18.5 ± 1.4; 5637, 37.0 ± 2.5; T24, 9.2 ± 1.5; and J82, 19.6 ± 2.1.

### *Cell counts*

To determine the effect of BCG on cell number, BCG was added to plated tumour cells for 72 h. Cells were washed twice with phosphate-buffered saline (PBS), detached with trypsin–EDTA and counted by 10% trypan blue (Flow Laboratories, Irvine, UK) exclusion using a Neubauer haemocytometer. Cell number and cell viability were determined.

### *Statistical methods*

The data were analysed using the Mann–Whitney *U*-test. Within each experiment (*n*) there were three replicate wells per treatment. The results were expressed as the mean of the percentage specific releases ± the standard error (s.e.). A regression analysis was performed for data which concerned a dose–response effect.

### Results

### *Effects of BCG alone*

The four target cell lines, UCRU-BL-17, 5637, T24 and J82, were incubated for 72 h at 37°C with 5, 50, 250 or 500 μg ml$^{-1}$ BCG. BCG increased the percentage specific release of [$^3$H]thymidine from the targets and for the T24 ($r^2 = 0.98$, $P = 0.0012$) and J82 ($r^2 = 0.912$, $P = 0.0113$) cell lines in a dose-dependent manner (Figure 1) ($r^2 = 0.82$, $P = 0.0343$, for UCRU-BL-17; $r^2 = 0.218$, $P = 0.5332$, for 5637). Decreasing significant *P*-values with increasing BCG were seen for the T24 cells (5–50 μg ml$^{-1}$, $P = 0.1747$; 5–250 μg ml$^{-1}$, $P = 0.0268$; and 5–500 μg ml$^{-1}$, $P = 0.0062$).

To confirm that the release of [$^3$H]thymidine represented a cytotoxic effect, we selected two cell lines UCRU-BL-17, which was sensitive to effector-mediated killing, and J82, which showed a dose-dependent direct response to BCG, and exposed them to BCG for 72 h to compare the cytotoxicity assay method with cell counts (Figure 2). For both cell lines, counts significantly decreased (for UCRU-BL-17 $P = 0.0374$ with BCG 250 μg ml$^{-1}$ and $P = 0.0163$ with BCG 500 μg ml$^{-1}$, $r^2 = 0.679$, $P = 0.1758$; for J82 $P = 0.0051$ with BCG 250 μg ml$^{-1}$ and $P = 0.0039$ with BCG 500 μg ml$^{-1}$, $r^2 = 0.862$, $P = 0.0716$) and percentage specific release increased (for UCRU-BL-17 $P = 0.0209$ with both 250 and 500 μg ml$^{-1}$ BCG, $r^2 = 0.789$, $P = 0.1116$; for J82 $P = 0.0209$ with 50, 250 and 500 μg ml$^{-1}$ BCG, $r^2 = 0.594$, $P = 0.229$) with increasing BCG concentration. We found no increase in the percentage of dead cells with increasing BCG at the concentrations tested, indicating that lysis was complete (data not shown).

To determine whether a donor's previous exposure to BCG made their immune cells more responsive to BCG in a cell culture system with UCRU-BL-17 target cells, effector cells were isolated from peripheral blood from healthy donors of different Mantoux status. We selected a group of six donors,

**Figure 2** Increasing BCG concentration caused an increase in the percentage specific release of incorporated [$^3$H]thymidine and a decrease in cell counts for UCRU-BL-17 (**a**) and J82 (**b**) (for UCRU-BL-17: for percentage specific release $P = 0.0209$ with BCG 250 and 500 μg ml$^{-1}$; for cell counts $P = 0.0374$ with BCG 250 μg ml$^{-1}$, $P = 0.0163$ with BCG 500 μg ml$^{-1}$; for J82: for percentage specific release $P = 0.0209$ for BCG 50, 250 and 500 μg ml$^{-1}$; for cell counts $P = 0.0051$ for BCG 250 μg ml$^{-1}$, $P = 0.0039$ for BCG 500 μg ml$^{-1}$) (*$P < 0.05$).

**Figure 3** Monocytes and lymphocytes isolated from Mantoux negative donors (1 – ve, 2 – ve), Mantoux-positive donors (3 + ve, 4 + ve) and donors who had had a BCG injection in the last 12 months (5 BCG, 6 BCG) were stimulated with 250 μg ml$^{-1}$ BCG. No difference was detected in the cytotoxicity of effectors from different donors towards UCRU-BL-17 target cells (– ve *vs* + ve, $P = 0.7488$; – ve *vs* BCG, $P = 0.7488$; + ve *vs* BCG, $P = 0.631$). The control was UCRU-BL-17 cells alone.

of whom two were Mantoux negative (– ve), two Mantoux positive (+ ve) and two had had a recent BCG injection following a negative Mantoux test (BCG). We found no correlation between *in vitro* effector response to BCG and donor exposure to BCG (– ve *vs* + ve; $P = 0.7488$; – ve *vs* BCG, $P = 0.7488$; + ve *vs* BCG, $P = 0.631$) (Figure 3).

Unstimulated lymphocytes had a significantly higher cytotoxic activity (19.6% ± 4.6%) than unstimulated monocytes/macrophages (6.8%–1.6%) ($P = 0.0041$) against the cell line UCRU-BL-17, and both were significantly higher than the spontaneous release by target cells alone (monocytes *vs* control $P = 0.0013$; lymphocytes *vs* control $P = 0.0001$) (Figure 4). This trend was observed for the 5637 and J82 cell lines but was not statistically significant (data not shown). As the UCRU-BL-17 cell line appeared to be more sensitive to effector-mediated killing, it was selected to test BCG- and cytokine-enhanced effector cytotoxicity. The cytotoxic activity of both monocytes/macrophages and lymphocytes against UCRU-BL-17 target cells was increased by pre-exposure to BCG (5, 50 or 250 $\mu g\ ml^{-1}$) (Figure 5). Monocyte/macrophage cytotoxicity was significantly enhanced by BCG in a dose-dependent fashion (from 7.8% ± 2.1% to 16.3% ± 2.4% with BCG 5 $\mu g\ ml^{-1}$, $P = 0.0093$; to 25.8% ± 3.0% with 250 $\mu g\ ml^{-1}$ and 25.9% ± 3.4% with 500 $\mu g\ ml^{-1}$, $P = 0.0001$ for both, $r^2 = 0.73$, $P = 0.1455$). Lymphocyte cytotoxicity increased at the higher concentrations (from 21.3% ± 4.4% to 30.6% ± 6.3% with 250 $\mu g\ ml^{-1}$ BCG, $P = 0.2517$, $r^2 = 0.869$, $P = 0.0676$). Results obtained from experiments with the 5637, T24 and J82 cell lines did not demonstrate a consistent trend (data not shown). When the filtered SNs of BCG-treated monocytes/macrophages or lymphocytes were added to UCRU-BL-17 target cells, the percentage specific release was significantly higher than that from targets incubated with SNs from untreated effectors ($P = 0.0209$ for each BCG concentration and incubation time for both effectors) (Figure 6). Similar significant differences were seen when using the J82 target line (data not shown).

**Figure 4** Unstimulated lymphocytes caused a significantly higher 72 h percentage specific release by target cells UCRU-BL-17 than unstimulated monocytes ($P = 0.0041$). The [$^3$H]thymidine release in the presence of both effector types was significantly higher than the spontaneous release by the control, UCRU-BL-17 target cells alone (monocytes, $P = 0.0013$; lymphocytes, $P = 0.0001$) (*$P < 0.05$).

To determine whether BCG pretreatment made target cells more susceptible to effector-mediated cytolysis, UCRU-BL-17 cells were pretreated for 48 h with BCG. Treated targets were no more susceptible than untreated targets to lysis by BCG- or cytokine-stimulated monocytes/macrophages or lymphocytes (data not shown).

Monocytes and lymphocytes were isolated from the peripheral blood of two pretherapy bladder cancer patients. The patients were male, 61 (patient 1) and 74 (patient 2) years old, and information on previous exposure to BCG was not available from their medical records. BCG-stimulated effector cells isolated from patient 2 but not from patient 1 (monocytes, $P = 0.009$; lymphocytes, $P = 0.0947$) (Figure 7).

*Effects of cytokines*

As the enhanced cytotoxicity in the presence of BCG appeared to be mediated by cytokine release, we tested the

**Figure 5** UCRU-BL-17 cells were co-cultured with BCG-treated monocytes (**a**) or lymphocytes (**b**) for 72 h. Monocyte/macrophage cytotoxicity towards UCRU-BL-17 cells was significantly enhanced by BCG ($P = 0.0093$ with 5 $\mu g\ ml^{-1}$ BCG; $P = 0.0001$ with 250 or 500 $\mu g\ ml^{-1}$ BCG). Lymphocyte cytotoxicity increased from 21.3% ± 4.4% to 30.6% ± 6.3% with 250 $\mu g\ ml^{-1}$ BCG ($P = 0.2517$) (*$P < 0.05$).

**Figure 6** When filtered SNs from monocytes/macrophages (**a**) and lymphocytes (**b**) were added to UCRU-BL-17 target cells, the target cell lysis was significantly greater if the effectors had been incubated with BCG ($P = 0.0209$). There was no difference between the incubation times (24, 48 or 72 h) or BCG doses (250 or 500 $\mu g\ ml^{-1}$) tested (*$P < 0.05$).

effects of specific cytokines on effector-mediated cytotoxicity. The cytokines IFN-α2b, IFN-γ and IL-2 at concentrations of 10, 100 or 1000 U ml$^{-1}$ were added to the tumour target cells alone (control) or with effectors. IFN-α2b and IL-2 caused an increase in percentage specific release by UCRU-BL-17 cells (for IFN-α2b: for an increase of 10 to 100 U ml$^{-1}$, $P = 0.5715$, 10 to 1000 U ml$^{-1}$, $P = 0.5119$, $r^2 = 0.675$, $P = 0.1783$; for IFN-γ: 10 to 100 U ml$^{-1}$, $P = 1$, 10 to 1000 U ml$^{-1}$, $P = 0.8046$, $r^2 = 0.068$, $P = 0.7391$; for IL-2: 10 to 100 U ml$^{-1}$, $P = 0.8977$, $r^2 = 919$, $P = 0.1836$), but this was not statistically significant (Table I). There was no observed increase for the other three cell lines (data not shown). IFN-α2b, IFN-γ and IL-2 did not significantly enhance monocyte/macrophage or lymphocyte killing of any of our cell lines at the doses tested (data not shown).

*Combinations of BCG and cytokines*

The effects of BCG combined with IFN-α2b, IFN-γ or IL-2 at the concentrations stated above were investigated using the four cell lines. In case the effects were due to timing, BCG and IFN-α2b were added together or sequentially, one 6 h after the other, in either order. We found neither evidence of supra-additivity nor any advantage in adding the biological response modifiers together as compared with separately in either order (data not shown).

*T-cell/natural killer (NK) cell cytotoxicity*

T cells and NK cells were compared with peripheral blood mononuclear cells (PBMCs) and lymphocytes in the above assay at 72 h and 144 h (Figure 8). The percentage specific release for all populations increased with time and BCG concentration. Addition of 250 μg ml$^{-1}$ BCG to effectors increased their 72 h percentage specific release as follows: control, 0% to 7.2%; PBMCs, 16.1% to 30.6%; lymphocytes, 11.7% to 25.4%; T cells, 4.4% to 6.3%; and T cells + 20% NK cells, 5.7% to 7.2%. These increases were not statistically significant ($P = 0.1732$). At 144 h the percentage specific release in all effector populations strongly correlated with BCG concentration (control, $r^2 = 0.727$, $P = 0.3499$; PBMCs, $r^2 = 1$, $P = 0.0066$; lymphocytes, $r^2 = 0.996$, $P = 0.0421$; T cells, $r^2 = 0.941$, $P = 0.1557$; and T cells + 20% NK cells, $r^2 = 0.998$, $P = 0.0275$).

### Discussion

BCG enhanced cytotoxic activity of monocytes and lymphocytes against bladder cancer cell lines *in vitro* and also had a small direct effect on these target cells. In four bladder tumour target lines, BCG mediated an increase in percentage specific release, accompanied by a decrease in cell numbers but not cell viability, suggesting an antiproliferative rather than a cytotoxic effect. BCG can bind in a dose-dependent manner to T24 cells (Mitzutani *et al.*, 1991) and is internalised by both mouse and human bladder tumour cells (Becich *et al.*, 1991). Although direct cytotoxicity of BCG or cytokines alone was not demonstrable against bladder cancer cell lines (Bohle *et al.*, 1993), it could result from release of bacterial components during BCG degradation or from proliferation of intracellular bacteria.

Previous exposure of a donor to BCG failed to enhance *in vitro* BCG-mediated effector cytotoxicity, but effectors from two pretherapy bladder cancer patients showed different responses. An understanding of the basis of patient response variation may help to predict who would benefit from BCG immunotherapy.

Monocytes and lymphocytes have been identified in immune infiltrates in the bladder following BCG administration (De Boer *et al.*, 1991*a*; Peuchmaur *et al.*, 1991) and an intact thymus-dependent immune response is required for the anti-tumour activity of BCG (Ratliff *et al.*, 1987; Ratliff, 1992). Flow cytometric analysis indicated that lymphocyte fractions used in our study contained T, B and NK cells and some polymorphonuclear (PMN) cells, while the monocyte population was more homogeneous (78% CD14$^+$). PBMCs and unsorted lymphocytes were more cytotoxic than sorted T cells or T cells + 20% NK cells, which showed a slower response, requiring over 144 h. Each effector group was more cytotoxic at 144 h than at 72 h. Others have shown that PBMCs incubated with BCG increase cytotoxicity to a maximum at 7 days (Bohle *et al.*, 1993) but are unable to demonstrate NK-mediated killing of bladder cancer cell lines.

The greater cytotoxic activity of unstimulated lymphocytes compared with unstimulated monocytes/macrophages may be due to cytokine release by effectors in the lymphocyte frac-

**Table I** Effect of cytokines on percentage specific release of [$^3$H]thymidine by UCRU-BL-17 bladder tumour cells

| *Cytokine* | *Concentration (U ml$^{-1}$)* 10 | 100 | 1000 |
|---|---|---|---|
| IFN-α2b ($n$ = 9–18) | 2.7 ± 1.0 | 4.0 ± 1.0 | 6.4 ± 2.4 |
| IFN-γ ($n$ = 6–14) | 3.0 ± 2.0 | 3.7 ± 1.5 | 3.1 ± 1.0 |
| IL-2 ($n$ = 12–14) | 2.5 ± 1.2 | 6.6 ± 3.2 | Not tested |

**Figure 7** Monocytes (circles) and lymphocytes (squares) isolated from the blood of two pretherapy bladder cancer patients (patient 1, open symbols; patient 2, closed symbols) were stimulated with BCG, but only those from one patient became more cytotoxic towards UCRU-BL-17 tumour cells (monocytes, $P = 0.009$; lymphocytes, $P = 0.0947$). As each sample was only available once, the results are expressed as the mean of triplicate wells in a single experiment (*$P < 0.05$).

**Figure 8** The percentage specific release by UCRU-BL-17 target cells co-cultured with PBMCs, lymphocytes, T cells and T cells + 20% NK cells increased with increasing BCG concentration and incubation time, but this was not statistically significant (0 *vs* 50 μg ml$^{-1}$ BCG, $P = 0.2568$; 0 *vs* 250 μg ml$^{-1}$ BCG, $P = 0.1041$; 72 h *vs* 144 h: no BCG, $P = 0.2101$; 50 μg ml$^{-1}$ BCG, $P = 0.4647$; 250 μg ml$^{-1}$ BCG, $P = 0.3472$. The results are expressed as the mean of triplicate samples in a single experiment.

tion. We found that SNs from BCG-activated monocytes/macrophages and lymphocytes increased target cell lysis, indicating that BCG cytotoxicity is largely cytokine mediated. Possible candidate cytokines include tumour necrosis factor alpha (TNF-α), IFN-γ, IL-1β and IL-6 (Kurisu *et al.*, 1995). This is supported by clinical studies which show that BCG therapy induces the presence of IL-1, IL-2, IL-6, TNF-α (De Boer *et al.*, 1992) and IFN-γ (Prescott *et al.*, 1990) in urine, probably derived from activated lymphocytes and, similarly, IFN-γ, IL-2, TNF-α and TNF-β are found in SNs of BCG-activated PBMCs *in vitro* (Thanhauser *et al.*, 1993). In other studies, $CD8^+/CD56^+$ lymphocytes, but not $CD4^+$ cells or macrophages, have been found to be responsible for BCG-induced cytolysis (Thanhauser *et al.*, 1993).

Both IFN-α2b and IL-2, but not IFN-γ, caused a small increase in the percentage specific release by UCRU-BL-17 cells. The mechanism underlying these effects was not clear, although IFN-α has proven effectiveness *in vivo* (Torti *et al.*, 1988). Others have shown that IL-2 at $10\ U\ ml^{-1}$ induces LAK activity against bladder cancer cell lines after 3 days, but that maximum activity requires 6 days with $1000\ U\ ml^{-1}$ (Jackson *et al.*, 1992). Tumour cells modified to express IL-2 and injected into tumour-bearing mice have been shown to give better anti-tumour effects than cisplatin or IFN-γ-producing cells against MBT2 mouse tumours, but no memory was established in 'cured' mice (Connor *et al.*, 1993). IL-2 also contributes to the maturation of effector cells and stimulates IFN-γ production, inducing activation of macrophages and cytotoxic T cells (Ikemoto *et al.*, 1990). The increase to maximum cytotoxicity by monocytes incubated with IL-2 occurs at 8–16 days (Higashi *et al.*, 1992). The assay time which we used may not be sufficient to demonstrate maximum IL-2-mediated increases in effector cytotoxicity.

In the assay used, BCG was more efficient than cytokines in stimulating effector cells alone. The response of macrophages has been shown to depend on the activating agent. Thus, lymphokine-triggered cytotoxic activity in bone marrow-derived phagocytes declines after 24 h, but that elicited by bacteria persists (Keller *et al.*, 1990). Furthermore, the secretion of IL-6 and prostaglandin $E_2$ ($PGE_2$) is enhanced by bacteria but not by lymphokines. In our experiments, a combination of BCG and cytokines was not supra-additive at the concentrations tested and an isobolographic analysis was not therefore justified (Goldstein *et al.*, 1989).

The bladder cancer lines used in this study differed in susceptibility to cytotoxic effects of BCG. Preincubation with BCG induced a dose-dependent increase in cytotoxic activity towards UCRU-BL-17, but not towards 5637, T24 or J82 bladder cancer cells. Others have also found that effector cell killing is independent of the histological grade of the parent tumour and the donor of the effector cells (Jackson *et al.*, 1992). Possible explanations for differences in target cell susceptibility relate to expression of cytokine receptors or tumour-associated antigens. The UCRU-BL-17 line has been established much more recently than the other cell lines, which may have undergone changes in resistance over their long period of tissue culture.

The mechanisms of BCG action are unclear but involve both direct effects possibly mediated via internalised bacteria and indirect immune effects involving immune infiltrates found in the bladder wall and urine of patients treated with BCG instillations. *In vitro*, the cytotoxic effect of monocytes/macrophages and lymphocytes is significantly enhanced by BCG, indicating that this could be an important indirect immune effect of BCG. Further work is needed to clarify the role of effector cells, using specific defined leucocyte subpopulations, and to understand the differential sensitivity of target cells to cytotoxic effects.

**Acknowledgements**
We would like to thank Dr Margaret Cooley for providing the flow cytometry expertise, and Drs Margaret Cooley and William Sewell for critical reading of the manuscript.

**References**

BECICH MJ, CARROLL S AND RATLIFF TL. (1991). Internalization of bacille Calmette–Guerin by bladder tumour cells. *J. Urol.*, **145**, 1316–1324.

BOHLE A, THANHAUSER A, ULMER AJ, ERNST M, FLAD HD AND JOCHAM D. (1993). Dissecting the immunobiological effects of bacillus Calmette–Guerin (BCG) in vitro: evidence of a distinct BCG-activated killer (BAK) cell phenomenon. *J. Urol.*, **150**, 1932–1937.

CONNOR J, BANNERJI R, SAITO S, HESTON W, FAIR W AND GILBOA E. (1993). Regression of bladder tumours in mice treated with interleukin 2 gene-modified tumour cells. *J. Exp. Med.*, **177**, 1127–1134.

DE BOER EC, DE JONG WH, STEERENBERG PA, VAN DER MEIJDEN APM, AARDEN LA, DEBRUYNE FMJ AND RUITENBERG EJ. (1991*a*). Leukocytes and cytokines in the urine of superficial bladder cancer patients after intravesical immunotherapy with bacillus Calmette–Guerin. *In Vivo*, **5**, 671–678.

DE BOER EC, DE JONG WH, VAN DER MEIJDEN APM, STEERENBERG PA, WITJES JA, VEGT PDJ, DEBRUYNE FMJ AND RUITENBERG EJ. (1991*b*). Presence of activated lymphocytes in the urine of patients with superficial bladder cancer after intravesical immunotherapy with bacillus Calmette–Guerin. *Cancer Immunol. Immunother.*, **33**, 411–416.

DE BOER EC, DE JONG WH, STEERENBERG PA, AARDEN LA, TETTEROO E, DE GROOT ER, VAN DER MEIJDEN APM, VEGT PDJ, DEBRUYNE FMJ AND RUITENBERG EJ. (1992). Induction of urinary interleukin-1 (IL-1), IL-2, IL-6, and tumour necrosis factor during intravesical immunotherapy with bacillus Calmette–Guerin in superficial bladder cancer. *Cancer Immunol. Immunother.*, **34**, 306–312.

DE JONG WH, DE BOER EC, VAN DER MEIJDEN APM, VEGT P, STEERENBERG PA, DEBRUYNE FMJ AND RUITENBERG EJ. (1990). Presence of interleukin-2 in urine of superficial bladder cancer patients after intravesical treatment with bacillus Calmette–Guerin. *Cancer Immunol. Immunother.*, **31**, 182–186.

DE WEGER RA, RUNHAAR BA AND OTTER WD. (1986). Cytotoxicity by macrophages and monocytes. In *Methods in Enzymology*. pp. 458–477. Academic Press: New York.

EL-DEMIRY MIM, HARGREAVE TB, BUSUTTIL A, JAMES K AND CHISHOLM GD. (1986). Immunohistochemical identification of lymphocyte subsets and macrophages in normal human urothelium using monoclonal antibodies. *Br. J. Urol.*, **58**, 436–442.

GOLDSTEIN D, BUCHMEYER SM, WITT PL, JORDAN VC AND BORDEN EC. (1989). Effects of type I and II interferons on cultured human breast cells: interaction with estrogen receptors and with tamoxifen. *Cancer Res.*, **49**, 2698–2702.

HIGASHI N, NISHIMURA Y, HIGUCHI M AND OSAWA T. (1991). Human monocytes in a long-term culture with interleukin-2 show a high tumoricidal activity against various tumour cells. *J. Immunother.*, **10**, 247–255.

IKEMOTO S, KISHIMOTO T, NISHIO S AND MAEKAWA M. (1990). Clinical studies on cell mediated immunity in patients with urinary bladder carcinoma: blastogenic response, interleukin-2 production and interferon-γ production of lymphocytes. *Br. J. Urol.*, **65**, 333–338.

JACKSON AM, HAWKYARD SJ, PRESCOTT S, RITCHIE AWS, JAMES K AND CHISHOLM GD. (1992). An investigation of factors influencing the in vitro induction of LAK activity against a variety of human bladder cancer cell lines. *J. Urol.*, **147**, 207–211.

KELLER R, KEIST R AND FREI K. (1990). Lymphokines and bacteria, that induce tumoricidal activity, trigger a different secretory response in macrophages. *Eur. J. Immunol.*, **20**, 695–698.

KOGA S, TANIGUCHI K, NISHIKIDO M, KUBOTA S, YAMASHITA S, KANETAKE H AND SAITO Y. (1991). Development of bacillus Calmette–Guerin-induced antitumour activity in peripheral blood mononuclear cells. *Urol. Int.*, **47**, 80–82.

KURISU H, MATSUYAMA H, OHMOTO Y, SHIMABUKURO T AND NAITO K. (1995). Cytokine-mediated antitumour effect of bacillus Calmette–Guerin on tumour cells in vitro. *Cancer Immunol. Immunother.* (in press).

LAMM DL. (1992). Optimal BCG treatment of superficial bladder cancer as defined by American trials. *Eur. Urol.*, **21** (Suppl. 2), 12–16.

LAMM DL, VAN DER MEIJDEN ADPM, MORALES A, BROSMAN SA, CATALONA WJ, HERR HW, SOLOWAY MS, STEG A AND DE-BRUYNE FMJ. (1992). Incidence and treatment of complications of bacillus Calmette–Guerin intravesical therapy in superficial bladder cancer. *J. Urol.*, **147**, 596–600.

MIZUTANI Y, NIO Y, FUKUMOTO M AND YOSHIDA O. (1992). Effects of bacillus Calmette–Guerin on cytotoxic activities of peripheral blood lymphocytes against human T24 lined and freshly isolated autologous urinary bladder transitional carcinoma cells in patients with urinary bladder cancer. *Cancer*, **69**, 537–545.

MORALES A, EIDINGER D AND BRUCE AW. (1976). Intracavitary bacillus Calmette–Guerin in the treatment of superficial bladder tumours. *J. Urol.*, **116**, 180–183.

PEUCHMAUR M, BENOIT G, VIEILLEFOND A, CHEVALIER A, LEMAIGRE G, MARTIN ED AND JARDIN A. (1989). Analysis of mucosal bladder leukocyte subpopulations in patients treated with intravesical Bacillus Calmette–Guerin. *Urol. Res.*, **17**, 299–303.

PRESCOTT S, JAMES K, HARGREAVE TB, CHISHOLM GD AND SMYTH JF. (1990). Radio-immunoassay detection of interferon-gamma in urine after intravesical Evans BCG therapy. *J. Urol.*, **144**, 1248–1251.

RATLIFF TL. (1992). Role of the immune response in BCG for bladder cancer. *Eur. Urol.*, **21**, 17–21.

RATLIFF TL, SHAPIRO A AND CATALONA WJ. (1986). Inhibition of murine bladder tumour growth by bacille Calmette–Guerin: lack of a role of natural killer cells. *Clin. Immunol. Immunopathol.*, **41**, 108–115.

RATLIFF TL, GILLEN D AND CATALONA WJ. (1987). Requirement of a thymus dependent immune response for BCG-mediated antitumour activity. *J. Urol.*, **137**, 155–158.

RUSSELL PJ, JELBART M, WILLS E, SINGH S, WASS J, WOTHERSPOON J AND RAGHAVAN D. (1988). Establishment and characterization of a new human bladder cancer cell line showing features of squamous and glandular differentiation. *Int. J. Cancer*, **41**, 74–82.

THANHAUSER A, BOHLE A, FLAD H-D, ERNST M, MATTERN T AND ULMER AJ. (1993). Induction of bacillus-Calmette–Guerin-activated killer cells from human peripheral blood mononuclear cells against human bladder carcinoma cell lines in vitro. *Cancer Immunol. Immunother.*, **37**, 105–111.

TORTI FM AND LUM BL. (1987). Superficial bladder cancer. Risk of recurrence and potential role for interferon therapy. *Cancer*, **59**, 613–616.

TORTI FM, SHORTLIFFE LD, WILLIAMS RD, PITTS WC, KEMPSON RL, ROSS JC, PALMER J, MEYERS F, FERRARI M, HANNIGAN J, SPIEGEL R, MCWHIRTER K AND FREIHA F. (1988). Alpha-interferon in superficial bladder cancer: a Northern California Oncology Group study. *J. Clin. Oncol.*, **6**, 476–483.